# ^1^H NMR based urinary metabolites profiling dataset of canine mammary tumors

**DOI:** 10.1038/s41597-022-01229-1

**Published:** 2022-03-31

**Authors:** Songyeon Lee, Byung-Joon Seung, In Seok Yang, Jueun Lee, Taewoong Ha, Hee-Myung Park, Jae-Ho Cheong, Sangwoo Kim, Jung-Hyang Sur, Geum-Sook Hwang, Hojung Nam

**Affiliations:** 1 School of Electrical Engineering and Computer Science, Gwangju Institute of Science and Technology (GIST), Gwangju, 61005 South Korea; 2 Department of Veterinary Pathology, College of Veterinary Medicine, Konkuk University, Seoul, 05029 South Korea; 3 Department of Biomedical Systems Informatics and Brain Korea 21 PLUS Project for Medical Science, Yonsei University College of Medicine, Seoul, 03722 South Korea; 4 Integrated Metabolomics Research Group, Western Seoul Center, Korea Basic Science Institute, Seoul, 03759 South Korea; 5 Department of Veterinary Internal Medicine, College of Veterinary Medicine, Konkuk University, Seoul, 05029 South Korea; 6 Department of Chemistry and Nano Science, Ewha Womans University, Seoul, 03760 South Korea

**Keywords:** Breast cancer, Metabolomics, Statistical methods

## Abstract

The identification of efficient and sensitive biomarkers for non-invasive tests is one of the major challenges in cancer diagnosis. To address this challenge, metabolomics is widely applied for identifying biomarkers that detect abnormal changes in cancer patients. Canine mammary tumors exhibit physiological characteristics identical to those in human breast cancer and serve as a useful animal model to conduct breast cancer research. Here, we aimed to provide a reliable large-scale metabolite dataset collected from dogs with mammary tumors, using proton nuclear magnetic resonance spectroscopy. We identified 55 metabolites in urine samples from 20 benign, 87 malignant, and 49 healthy control subjects. This dataset provides details of mammary tumor-specific metabolites in dogs and insights into cancer-specific metabolic alterations that share similar molecular characteristics.

## Abstract (summary)

**Table:** 

Measurement(s)	Metabolite
Technology Type(s)	1H nuclear magnetic resonance spectroscopy
Factor Type(s)	Age • Breeds • Sex • ER status • HER2 status • Histological grade • Neutral status
Sample Characteristic - Organism	Canis lupus familiaris

## Background & Summary

According to the American Cancer Society, breast cancer ranks first in the incidence rate in women^1^. Although breast cancer has a higher survival rate than other types of cancer, early and accurate diagnosis is essential. Among various diagnostic tools for breast cancer, mammography has remained the gold standard. However, it poses the risk of exposure to radioactive material and involves high false-positive rates^2–4^, especially in benign stages during which non-cancerous cells show characteristics similar to those in malignant cells, obscuring diagnosis. To overcome these constraints, ongoing research has sought to identify biomarkers reflective of the physiological states of the disease, and of use in differentiating between normal and cancer specimens^5^. Such biomarkers are expected to facilitate steady monitoring of cancer and provide a better understanding of cancer phenomena. However, the identification of efficient and sensitive biomarkers requires a sufficiently large number of samples.

Owing to the lack of human samples, and the prevalence of ethical issues, animal models are commonly used to confirm drug efficacy and stability before clinical trials in humans^6^. Canine mammary tumors (CMTs) are the second most common neoplasias in dogs, and they exhibit similar clinical and molecular characteristics to breast cancer, including onset age, course of the disease, and estrogen dependency^7,8^. In this regard, we recently elucidated cross-species oncogenic signatures obtained from pet dogs as reference points for comparative analysis with human cancers for developing novel diagnostic and therapeutic technologies. As confirmed in a previous study, there is a striking resemblance of genomic characteristics, including frequent *PIK3CA* mutations (43.1%), aberrations of the PI3K-Akt pathway (61.7%), and key genes involved in cancer initiation and progression^9,10^. Here, we aimed to provide reliable large-scale proton nuclear magnetic resonance (^1^H NMR) spectroscopy data for cancer research on dogs.

**Fig. 1:**
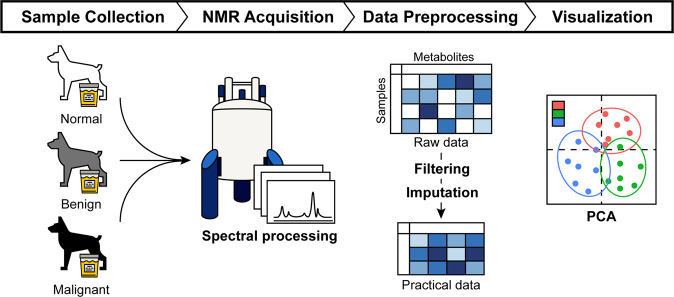
Overview of the experimental process in canine mammary tumors (CMTs). Urine samples were collected from healthy dogs and dogs with CMTs. The metabolites in urine were quantified using proton nuclear magnetic resonance (^1^H NMR) spectroscopy. The raw data were filtered and imputed to make complete datasets. For the data visualization, principal component analysis (PCA) was performed on the preprocessed data.

In this study, we obtained a large number of urine samples from dogs diagnosed with benign and malignant tumors, and also from healthy controls for detailed analysis of tumors and their effects. We then utilized NMR to investigate metabolites in the different grades of samples. The overall process described in this study is summarized in Fig. 1.

The urinary metabolite dataset from a large-scale canine cohort provides potential advantages for further research. To the best of our knowledge, this is the first study to describe a metabolomics dataset containing a large number of urine samples from dogs with mammary tumors and healthy controls. Despite the prevailing differences between breeds of dogs included in the dataset, their diversity will help cover the general characteristics of CMTs. The inclusion of both benign samples and highly divided tumor grades enables the efficient identification of biomarkers for the early detection of cancer. As mentioned earlier, the canine model is a good animal model for human disease, as it can be used for cross-species research that applies to the discovery of human breast cancer biomarkers.

## Methods

### Sample collection

Urine samples were collected from 117 dogs with CMTs and 49 healthy dogs. Urine specimens from the dogs with CMTs obtained as a part of routine screening tests (urine analysis or pre-anesthetic test) before surgery under fasting conditions from client-owned dogs via private veterinary clinics with informed consent. Only available cases diagnosed with CMT based on histological assessment were included in the study. Dogs with CMT received surgical treatment without adjuvant chemotherapy. Healthy control urine samples were obtained from surplus urine samples collected from client-owned healthy dogs or a healthy research beagle during a regular medical check-up. Only dogs with no history or clinical signs of tumors or any other disease were included as controls. Urine samples were collected by free catch or cystocentesis (surplus samples from the regular test). Residual samples were refrigerated at 4 °C for no longer than 24 h, frozen at −20 °C, transferred to −80 °C, and stored for up to 2 y before analysis. Stored residual samples were used in the current study and the procedures were conducted in accordance with the guideline of the Institutional Animal Care and Use Committee of Konkuk University (KU18168).

### Clinical sample information

All characteristics of the samples used in this study are summarized in Table 1. Because urine samples can be disturbed by other downstream metabolisms, we excluded samples that had concurrent tumors at other organ sites to avoid the influence of irrelevant tumors. Urine samples from 156 female dogs were retained, of which, 20 had benign and 87 had malignant CMTs; the remaining 49 dogs were used as healthy controls. Histological subtyping and grading of benign and malignant CMT samples were assessed as described in a previous publication^10^. Malignant samples were separated into three grades: grade 1 (early-stage cancer) was highly prevalent in 47 samples, whereas grades 2 and 3 had the same number of samples (20 each). Both benign and malignant subjects frequently showed positive expression of estrogen receptor (ER) and human epidermal growth factor receptor 2 (HER2). Information about the breeds included in each category is provided in Table 2.

**Table 1:** Clinical information of the canine mammary tumors and normal samples.

Characteristics	Benign	Malignant	Normal
Number	20	87	49
Age (month, mean ± SD)	115.33 ± 31.53	150.28 ± 33.18	43.04 ± 31.71
Histological grade			
1	N/A	47	N/A
2		20	
3		20	
ER			
+	19	68	N/A
−	1	14	
HER2			
+	16	62	N/A
−	4	20	
Neutral status			
Intact	17	51	16
Neutered	3	33	24

The samples without clinical information were not included in the counts.

SD, Standard deviation; N/A, not available; ER, estrogen receptor; HER2, human epidermal growth factor receptor 2.

**Table 2:** Breed information for canine mammary tumors and healthy control subjects.

Breeds	Benign	Malignant	Normal
Beagle	0	1	17
Bichon Frise	0	0	1
Chihuahua	0	3	2
Cocker Spaniel	1	5	1
Dachshund	2	1	0
Golden Retriever	0	0	1
Italian Greyhound	0	0	2
Maltese	7	32	11
Miniature Pinscher	0	3	0
Miniature Poodle	0	0	2
Mixed	1	9	1
Pekingese	0	0	0
Pomeranian	1	5	1
Poodle	3	8	2
Pug	0	0	1
Schnauzer	1	2	1
Shetland Sheepdog	0	0	1
Shih-tzu	1	7	2
Yorkshire Terrier	3	8	3
N/A	0	3	0

N/A, Not available.

### NMR sample preparation and acquisition

To quantify urinary metabolites from dogs with CMTs and healthy controls, ^1^H NMR-based metabolic profiling was performed using one urine sample from each dog. The experimental methods used for NMR analysis have been published elsewhere^11^. After thawing at 4 °C overnight, proteins in urine were removed with cut-off membrane filters (Amicon Ultra, 3 K, Merck Millipore, Middlesex County, MA, USA) by centrifugation at 18,213 × g and 4 °C for 20 min. Next, 330 μL of filtered urine was mixed with 350 μL of 0.2 M deuterated sodium phosphate buffer (pH 7.0). Deuterated sodium phosphate buffer, NMR solvent, was prepared by mixing 0.2 M sodium phosphate monobasic (Sigma-Aldrich, St. Louis, MO, USA) in deuterium oxide and 0.2 M sodium phosphate dibasic (Sigma-Aldrich) in deuterium oxide. The pH of the NMR solvent was then adjusted to 7.0 ± 0.1. After adjusting the pH to 7.0 ± 0.1 for each urine sample, a total of 630 μL of urine sample was mixed with 70 μL of deuterium oxide containing 5 mM TSP-d4 (3-(trimethylsilyl) propionic 2,2,3,3-d4 acid sodium salt (98% atom %). A total of 17.23 mg of TSP-d4 was dissolved in 20 mL of deuterium oxide to prepare 5 mM of TSP-d4. Finally, 600 μL of each sample was transferred into a disposable 5-mm NMR tube (Part No. Z112273, Bruker BioSpin, Rheinstetten, Germany). All NMR samples were handled before or after the NMR measurement on Sample Jet (Bruker BioSpin), which is an automated device. Prior to NMR measurement, urine samples were maintained at 4 °C in the Sample Jet and equilibrated at 25 °C for 3 min.

All one-dimensional proton (1D ^1^H) experiments were carried out at 25 °C on a Bruker Avance III HD 800-MHz NMR spectrometer (Bruker BioSpin) equipped with a Bruker 5-mm CPTCI Z-GRD probe. The NOESY PRESAT pulse sequence with water presaturation was used to acquire ^1^H NMR spectra collected into 65,536 data points, with 64 transients, a relaxation delay of 2.0 s, a spectral width of 20 ppm, and an acquisition time of 2.0 s. A 0.3-Hz line broadening function was applied to all spectra before Fourier transformation.

### Spectral processing

**Fig. 2:**
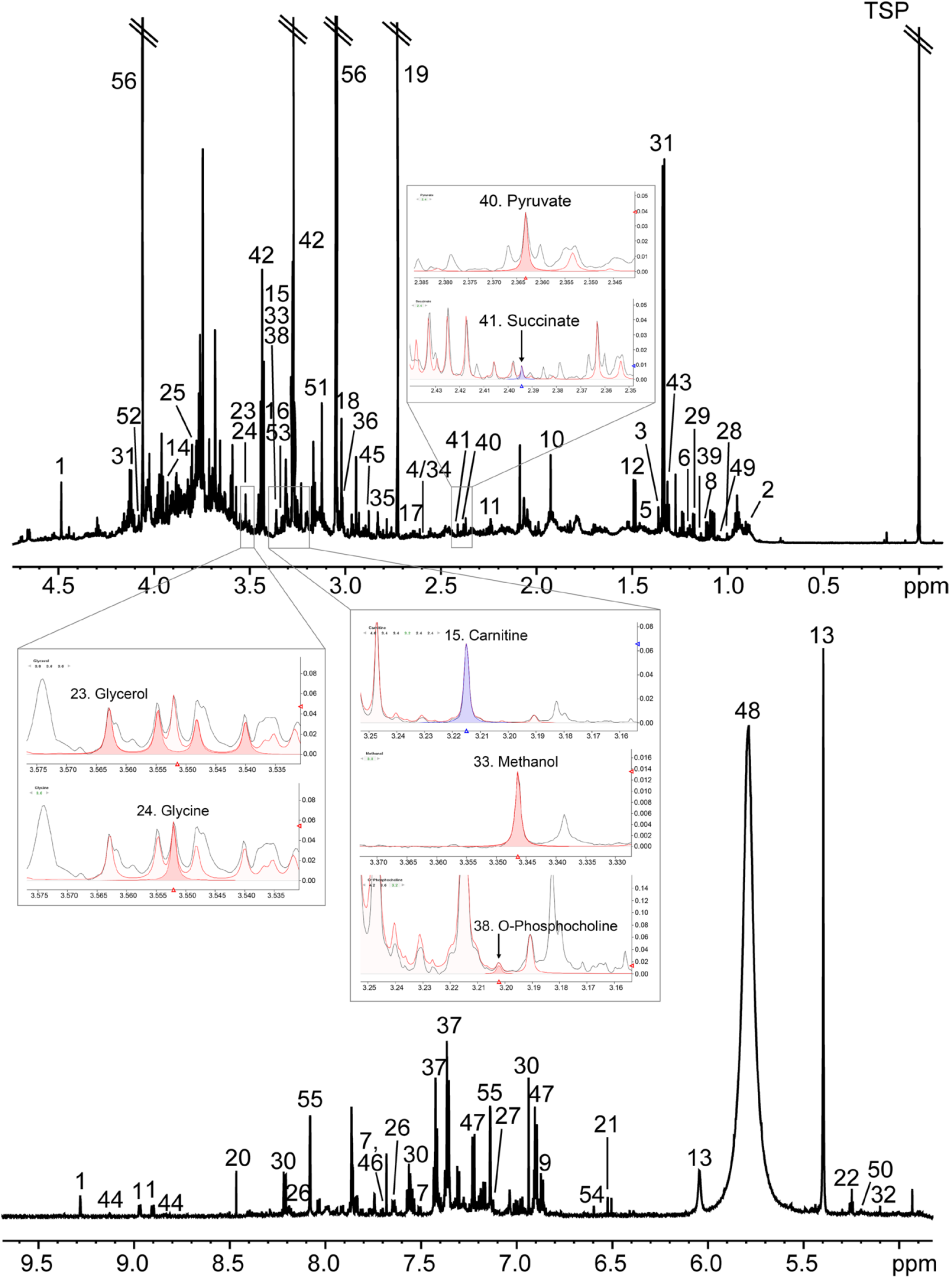
Representative 800 MHz proton nuclear magnetic resonance spectrum of dog urine with metabolite assignments and peak area integration for urinary metabolites. This NMR spectrum was derived from a dog (random number 8, sample ID 16K-271). Glycerol and glycine were assigned at 3.555 (doublet of doublet) and 3.552 (singlet) ppm, respectively. Carnitine, methanol, and O-phosphocholine were assigned at 3.215 (singlet), 3.346 (singlet), and 3.201 (singlet) ppm, respectively. Pyruvate and succinate were assigned at 2.364 (singlet) and 2.395 (singlet) ppm, respectively. The filled area colored with red or blue was the peak integration area for each metabolite. 1. 1-Methylnicotinamide; 2. 2-Hydroxybutyrate; 3. 2-Hydroxyisobutyrate; 4. 2-Oxoisocaproate; 5. 2-Phenylpropionate; 6. 3-Hydroxyisovalerate; 7. 3-Indoxylsulfate; 8. 3-Methyl-2-oxovalerate; 9. 4-Hydroxyphenylacetate; 10. Acetate; 11. Acetone; 12. Alanine; 13. Allantoin; 14. Betaine; 15. Carnitine; 16. Choline; 17. Citrate; 18. Creatine; 19. Dimethylamine; 20. Formate; 21. Fumarate; 22. Glucose; 23. Glycerol; 24. Glycine; 25. Guanidoacetate; 26. Hippurate; 27. Histidine; 28. Isoleucine; 29. Isopropanol; 30. Kynurenate; 31. Lactate; 32. Mannose; 33. Methanol; 34. Methylamine; 35. Methylguanidine; 36. N,N-Dimethylglycine; 37. N-Phenylacetylglycine; 38. O-Phosphocholine; 39. Propylene glycol; 40. Pyruvate; 41. Succinate; 42. Taurine; 43. Threonine; 44. Trigonelline; 45. Trimethylamine; 46. Tryptophan; 47. Tyramine; 48. Urea; 49. Valine; 50. Xylose; 51. cis-Aconitate; 52. myo-Inositol; 53. sn-Glycero-3-phosphocholine; 54. trans-Aconitate; 55. pi-Methylhistidine; 56. Creatinine.

All acquired spectra were phased and baseline corrected using the TopSpin (version 3.1, Bruker BioSpin) and AMIX (version 3.9.7, Bruker BioSpin) software, respectively. Afterward, urinary metabolites were identified and quantified using Chenomx NMR Suite (version 7.1, Chenomx Inc., Edmonton, Canada). TSP-d4 was used as a chemical shift reference with a value of −0.016 ppm. Fifty-six metabolites were identified and confirmed using the 800 MHz library of Chenomx, 2D NMR spectra, and spiking experiments. A representative ^1^H NMR spectrum of dog urine is shown in Fig. 2. The concentrations of 56 metabolites were assessed by integrating peak areas of metabolites in comparison with the areas of the 5 mM TSP peak. The examples of peak assignment and peak area integration for several metabolites in urine samples were attached on the corresponding ppm areas shown in Fig. 2. Finally, the levels of each urinary metabolite were normalized and adjusted with creatinine concentration.

### Statistical analysis

All missing values in raw data were imputed by three groups and evaluated using MetImp 1.2^12,13^ after excluding metabolites based on the modified 80% rule^14^. To apply the most proper imputation method for metabolomics data, we tested four types of imputation algorithms: an iterative Gibbs samplerbased left-censored missing value imputation approach (GSimp), quantile regression imputation of left-censored data (QRILC), half minimum (HM), and zero imputation method. To validate the performance of each imputation method, we extracted the complete dataset that eliminated all missing values from the original data and randomly generated approximately 5–40% of missing values in the dataset. We then selected the method with the lowest normalized root mean squared error and the Procrustes sum of squared error which represent how well the methods could recover the missing values against the complete data with the minimum differences.

The CMT samples were separated into benign and malignant specimens for comparison with normal samples. To visualize the distribution of the samples, log_2_ transformation was performed to make skewed distributions more symmetric^15^ and principal component analysis (PCA) was used in R.

## Data Records

The dataset supporting the conclusions of this article is available in the MetaboLights repository^16^ MTBLS2550 (https://www.ebi.ac.uk/metabolights/MTBLS2550^17^).

## Technical Validation

All samples were rearranged in random order and most experiments such as sample preparation, data acquisition, and data processing were accomplished in this order to avoid any artificial variations. During sample preparation, all samples were kept on ice to avoid metabolite degradation. The pH of the samples was finely adjusted to minimize variation in chemical shift among samples (7.0 ± 0.1). After sample preparation, all samples were kept at 4 °C in the autosampler rack before NMR data acquisition.

**Fig. 3:**
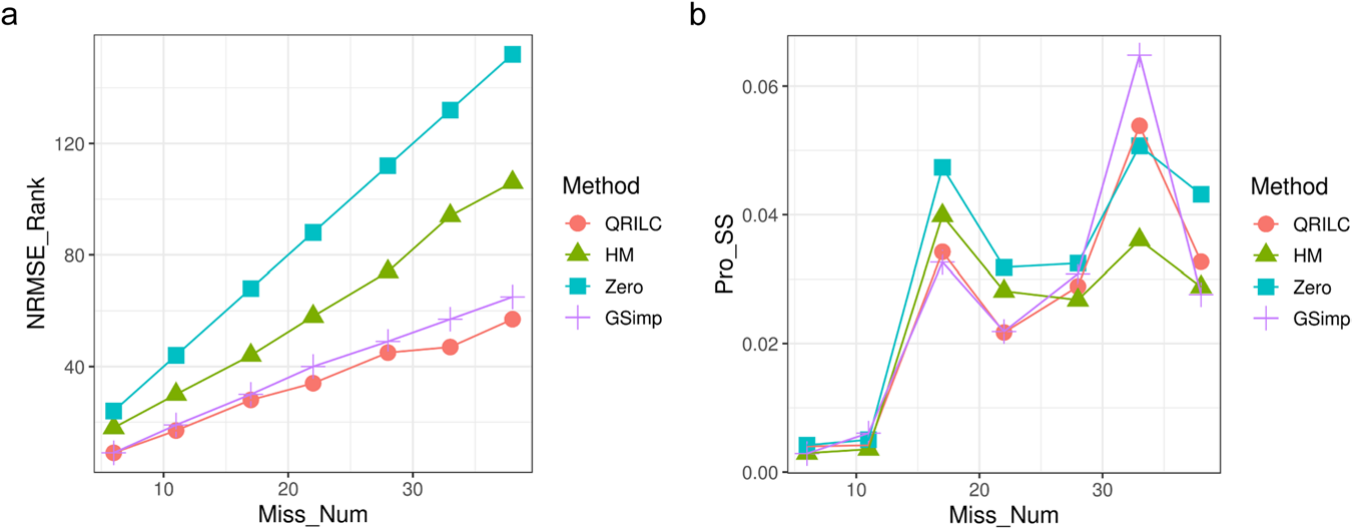
Performance evaluation of missing value imputation methods. Four imputation methods were evaluated along with the percentage of missing numbers (x-axis). (**a**) Normalized root mean squared error ranking plot. (**b**) Procrustes sum of squared error plot. QRILC, quantile regression imputation of left-censored data; HM, half minimum; GSimp, an iterative Gibbs sampler based left-censored missing value imputation approach.

After spectral processing, fumarate and citrate containing more than 20% of missing values in each group were eliminated. Among the four imputation methods, QRILC showed robust performance in the different proportions of missing values (Fig. 3a,b). As a result, the remaining missing values in the raw data were replaced by QRILC.

**Fig. 4:**
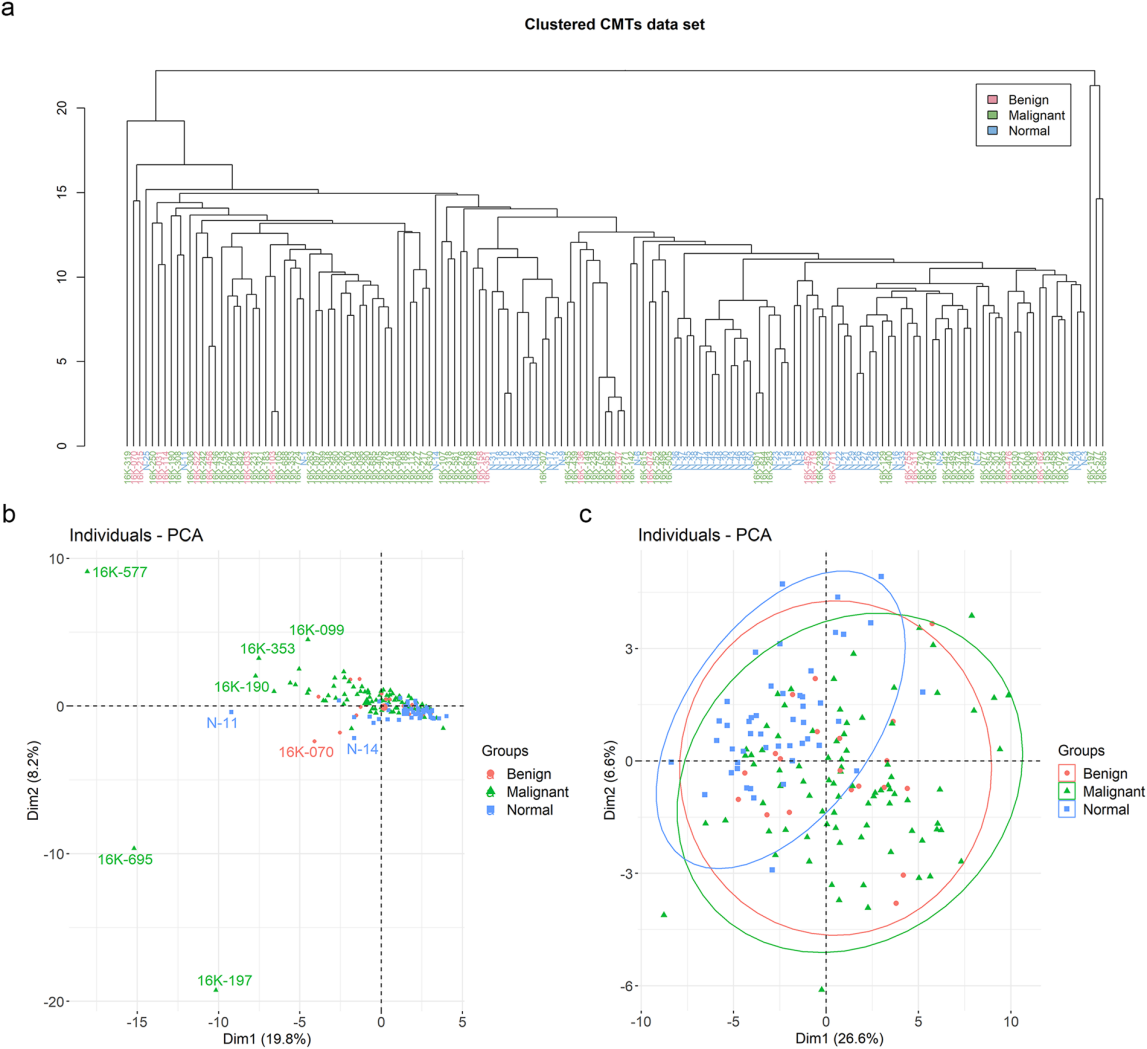
Hierarchical clustering with dendrogram and principal component analysis (PCA) plots. Red: benign, green: malignant, blue: normal subjects. (**a**) Dendrogram of 156 samples. (**b**) PCA plot of the raw data. The points on the plot represent the samples. (**c**) PCA plot of the preprocessed data after filtering the outlier samples.

To identify the outliers and visualize the distribution of the dataset, we drew the dendrogram and PCA score plot colored by groups. In Fig. 4a, three malignant samples stood off from the group clearly and one normal subject was far from the healthy control cluster in Fig. 4b. After eliminating four samples, we redrew the PCA score plot and confirmed that the samples were less biased (Fig. 4c).

**Fig. 5:**
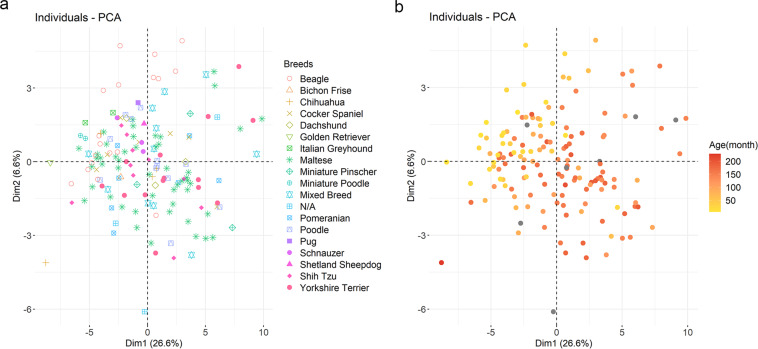
Principal component analysis (PCA) plots colored with factors after preprocessing. (**a**) PCA plot colored by breeds. (**b**) PCA plot colored by age. The unit of age is month and darker color indicates older subject. The samples having no age information are colored gray.

Furthermore, to validate the potential effect of minor breeds, and to check whether they will affect further statistical analysis by contributing to the noise, we checked the breeds of the outliers and colored the samples according to the breeds. The breeds of three outliers in the malignant group and a sample in the healthy control group were maltese, schnauzer, poodle, and chihuahua, respectively, which were not unique breeds in the dataset. In addition, various breeds in the dataset were spread widely (Fig. 5a). Comprehensively looking at these results, the dataset implied a weak effect of breed-specific characteristics for reproducibility. Lastly, we colored the points in the PCA plot by age in Fig. 5b to confirm whether age contributes to bias in the dataset.

## Usage Notes

In this study, we described the preprocessed metabolite dataset extracted from CMTs using ^1^H NMR. Although we excluded samples that contained concurrent tumors in other organs to prevent the impurity of metabolites, the information about these samples can be found in MetaboLights, along with the clinical notes.

The metabolites in the urine samples from the dogs with mammary tumors and healthy controls were normalized with creatinine which was considered as a standard method. However, there are some cases where creatinine normalization is not suitable, such as when the samples were not ensured the required creatinine clearance due to metabolic dysregulation^18^. Even though this dataset is not directly related to metabolic dysregulation diseases, researchers can explore the raw ^1^H NMR profiling data without creatinine normalization for flexible usage.

Client-owned dogs with CMTs were housed in a family environment, and not in a controlled environment, which made it difficult to control for all other possible environmental factors. However, results from client-owned dogs with spontaneous cancer might more closely reflect reality. Besides, there were differences in age and breed between the sample groups as summarized in Tables 1 and 2. The number of maltese dogs in the malignant group (32) was larger than in other groups, which is consistent with the data regarding the most common dog breeds owned by South Koreans. Still, the age of dogs with mammary tumors was similar to the median onset age of mammary tumors in canines, which is approximately 10.5 years, comparable to our dataset^8^. However, their average age is three times more than that of healthy controls. As previously mentioned, healthy control samples were collected from client-owned healthy dogs or a healthy research beagle, making it hard to match the age of the control group with the test groups. To deal with feature imbalances, researchers should perform the sampling while considering the ratio of other factors.

## Data Availability

The imputation of missing values in the raw ^1^H NMR metabolite concentration data can be accessed using the following URL: https://metabolomics.cc.hawaii.edu/software/MetImp/. The evaluation of each method used can be accessed on GitHub (https://github.com/WandeRum/MVI-evaluation)^12^. The R scripts for preprocessing of metabolites profile dataset and visualization are provided in the GitHub repository (https://github.com/GIST-CSBL/CMTs).
